# Association of Vegetables-Fruits Dietary Patterns with Gestational Diabetes Mellitus: Mediating Effects of Gut Microbiota

**DOI:** 10.3390/nu16142300

**Published:** 2024-07-17

**Authors:** Xiaoxi Shan, Caixia Peng, Hanshuang Zou, Yunfeng Pan, Minchan Wu, Qingqing Xie, Qian Lin

**Affiliations:** Department of Nutrition Science and Food Hygiene, Xiangya School of Public Health, Central South University, 172 Tongzipo Road, Changsha 410006, China; shanxiaoxi@csu.edu.cn (X.S.); pengcaixia@csu.edu.cn (C.P.); zouhanshuangcsu@foxmail.com (H.Z.); zndx_panyunfeng@csu.edu.cn (Y.P.); wuminchan@csu.edu.cn (M.W.); xieqingqing@csu.edu.cn (Q.X.)

**Keywords:** gestational diabetes mellitus, dietary patterns, gut microbiota, mediation analysis, pregnant women

## Abstract

(1) Introduction: Previous studies have found that diet can change gut microbiota, thereby affecting metabolic health. However, research on gestational diabetes mellitus (GDM) is still limited. Our study aimed to explore the mediating role of gut microbiota in the relationship between dietary patterns and GDM. (2) Methods: In this case-control study, 107 women with GDM at 24–28 weeks of gestation and 78 healthy pregnant women were enrolled. A semi-quantitative food frequency questionnaire (FFQ) was used to assess dietary intake over the previous month. Mediation analysis was performed to explore the link between dietary patterns, gut microbiota, and GDM. (3) Results: Among the five dietary patterns extracted, the high group (factor scores ≥ −0.07) of the vegetables-fruits dietary pattern had a 67% lower risk of developing GDM compared to the low group (factor scores < −0.07) (OR: 0.33; 95% CI: 0.15–0.74). In addition, a significant alteration was observed in gut microbiota composition among GDM pregnant women. Mediation analysis showed that the Lachnospiraceae family, *Blautia*, and *Ruminococcus* genus partially mediated the effect of vegetables-fruits dietary pattern on GDM, explaining 45.81%, 44.33%, and 31.53% of the association, respectively. (4) Conclusions: Adherence to vegetables-fruits dietary patterns during pregnancy may reduce the risk of GDM by altering gut microbiota composition.

## 1. Introduction

Gestational diabetes mellitus (GDM) is defined by WHO as varying degrees of impaired glucose tolerance that occurs or is first detected during pregnancy, and it is one of the most common metabolic disorders and complications during pregnancy [1]. Due to differences in populations and diagnostic criteria, the prevalence of GDM varies across countries, ranging from 6.6% to 45.3% [2]. In mainland China, the prevalence of GDM is approximately 14.8% when evaluated using the International Association of Diabetes and Pregnancy Study Groups (IADPSG) diagnostic criteria [3]. As an important public health issue, GDM can have detrimental effects on the health of both mothers and their offspring, including polyhydramnios, infections, preterm birth, macrosomia, and neonatal hypoglycemia [4].

The onset and progression of GDM are influenced by several factors, with dietary factors being particularly noteworthy [5]. Bao et al. revealed a positive correlation between pre-pregnancy potato consumption and the risk of developing GDM [6]. Additionally, several studies [7,8,9,10] have consistently demonstrated that consuming foods high in saturated fatty acids, such as animal meat and fast food, disrupted glucose homeostasis and subsequently increased the risk of GDM among pregnant women.

In recent years, there has been a growing focus on investigating the connection between dietary patterns and GDM, with an emphasis on taking into consideration the interaction of various food components [11]. A study showed that high adherence to the Mediterranean diet was associated with a lower incidence of GDM [12]. Furthermore, pregnant women who adopt a dietary pattern characterized by high dietary fiber intake had a negative correlation with the risk of GDM [13]. A dietary pattern rich in fruits and dairy products was a protective factor against GDM [14]. Conversely, it has been previously established that Western dietary patterns featuring refined grains, red meat, and fast food are linked to a higher risk of GDM [15,16]. However, dietary habits are influenced by regional and cultural factors. For instance, in China, traditional dietary beliefs encourage pregnant women to consume more meat and meat soup, which potentially increases their intake of saturated fatty acids. The dietary patterns of pregnant women extracted from studies conducted in different regions may exhibit variations, leading to a lack of definitive consensus regarding the relationship between dietary patterns and GDM.

Recently, there has been some emerging evidence indicating that the gut microbiota is involved in glucose and lipid metabolism, as well as inflammation and immune responses [17,18,19]. Compared to pregnant women with normal blood glucose levels, changes in the composition of gut microbiota have been observed in pregnant women with GDM [20,21,22,23]. Pregnant women with GDM usually have elevated Gram-negative bacteria and lipopolysaccharides (LPS) in the gut, which increases intestinal permeability, and a large amount of endotoxin enters the circulating bloodstream, stimulating an inflammatory response in the body. Prolonged low-grade inflammation affects the structure and function of pancreatic islet B-cells, leading to insulin resistance, which in turn triggers GDM [24,25]. However, findings lack consistency in the diversity and abundance of gut microbiota. Furthermore, previous studies have consistently indicated that dietary patterns could influence the gut microbiota [26,27,28]. Despite the fact that diet can alter the gut microbiota and subsequently affect metabolic health [29], research on GDM is still limited.

To our knowledge, few studies have investigated the relationship between dietary patterns, gut microbiota, and GDM. Given the significant health risks associated with GDM for both pregnant women and their fetuses, understanding these interactions is critical. Therefore, we conducted a case-control study aiming to identify the differences in dietary patterns and gut microbiota between women with GDM and those without GDM and to explore the relationship between these dietary pattern differences and gut microbiota differences. We hypothesized that the gut microbiota is a key mediating factor in dietary patterns affecting GDM.

## 2. Materials and Methods

### 2.1. Study Design and Participants

This study is a case-control study, including 107 pregnant women newly diagnosed with GDM and 78 healthy controls. The study was conducted in Changsha Maternal and Child Health Hospital from March to July 2022.

According to the standard set by the IADPSG, a 75 g oral glucose tolerance test (OGTT) was conducted between the 24th and 28th week of pregnancy to diagnose GDM. GDM could be confirmed if any of the following conditions are met: fasting blood glucose level ≥ 5.1 mmol/L, 1 h postprandial blood glucose level ≥ 10.0 mmol/L, or 2 h postprandial blood glucose level ≥ 8.5 mmol/L.

Women in the case group were recruited based on the following inclusion criteria: (1) pregnant women aged between 20 and 40 years; (2) at 24–28 gestational weeks; (3) the diagnosis of GDM was confirmed by 75g OGTT; (4) residing in Changsha for more than 12 months; (5) singleton pregnancy.

The controls were healthy pregnant women at 24–28 gestational weeks who tested negative for GDM.

Exclusion criteria for participants in both groups: (1) those who have undergone assisted reproductive techniques such as in vitro fertilization or artificial insemination; (2) those with a medical history encompassing diabetes, hypertension, thyroid disease, inflammatory bowel disease, asthma, or other related conditions; (3) pregnant individuals who have previously taken probiotics or antibiotics during the gestational period; (4) individuals displaying suboptimal compliance; (5) pregnant women affected by mental illness, communication impairments, or comprehension disorders.

### 2.2. Sample Size

The PASS software (version 15.0, NCSS, LLC, Kaysville, UT, USA) was used to calculate the sample size. Based on similar findings [30], it was assumed that 33% of the study participants in the control group adopted healthier dietary patterns before pregnancy (P_0_ = 0.33). It was expected that healthier dietary patterns would reduce the risk of GDM by 71.6% (OR = 0.284). With a significance level of 0.05 and a power of 80%, it was estimated that the minimum sample size was 61 participants in each group. Assuming a non-response rate of 20% among the participants, the sample size for both the case and control groups was calculated as 77.

### 2.3. Measurements

#### 2.3.1. Dietary Pattern

This study used a semi-quantitative Food Frequency Questionnaire (FFQ) to retrospectively collect dietary data of pregnant women in the past month. The FFQ questionnaire consists of three parts: food categories, consumption frequency, and average portion size per eating meal. The FFQ consisted of 10 major food categories, including cereals and tubers, meat products, aquatic products, eggs and dairy products, beans, vegetables and mushrooms, fresh fruits, snacks and nuts, beverages, seasonings, and cooking oils. Based on the characteristics of the food and actual investigation results, the food items in the FFQ were divided into 13 food groups, which contained beverages, snacks, processed meat products, poultry, animal meats, aquatic products, beans, mushrooms and seaweeds, eggs, cereals and tubers, milk and dairy products, fruits, vegetables. The pregnant women were interviewed by trained investigators to inquire about the frequency of food consumption for each item and the average intake. Frequency options include times per day, times per week, times per month, and food quantity is measured in grams. Food models, food diaries, and standardized tableware were used to estimate the average intake amount each time.

Dietary information collected by FFQ is translated into average daily intake. As the most commonly used method in dietary pattern research, factor analysis was used to construct the dietary patterns in this study. The KMO test (0.560) and Bartlett’s test of sphericity (*p* < 0.001) indicated that factor analysis was appropriate. Varimax rotation was employed to enhance interpretability. To enhance the informativeness and explanatory power of the extracted dietary patterns, we combined the following criteria: eigenvalues > 1, scree plot, cumulative variance contribution, and the interpretability of the dietary patterns. The rotated factor loading matrix was shown in Table S1, and food groups with factor loadings above an absolute value of 0.4 were used to name the dietary patterns. The calculation of dietary pattern factor scores was based on the intake of food groups under various dietary patterns, weighted according to their factor loadings. Each participant will have a factor score for each extracted dietary pattern, and a higher score for a specific pattern indicates a greater adherence to that particular dietary pattern among the study participants. In this study, the factor scores for each participant were presented in Table S2.

#### 2.3.2. Fecal Microbiota

Stool samples were self-collected by the participants. Briefly, the researchers provided the participants with detailed instruction manuals on how to collect the sample on their own. All materials were provided to the participant in a convenient insulated box with an ice pack. Sterile stool collection tubes with scoops were used by pregnant women to collect at least 0.5 g of fresh stool. After collection, stool samples were placed in the box and handed over to the researcher, who transported the samples to the laboratory at low temperatures and stored them in a refrigerator at −80 °C for 2 h. After the completion of fecal sample collection from all participants, the samples were transported under strict cold-chain conditions to Wekemo Tech Co., Ltd., Shenzhen, China, for subsequent comprehensive analysis and evaluation. Sequencing was performed on the Illumina platform (San Diego, CA, USA). 

#### 2.3.3. Covariates

Demographic variables were self-reported, including age, ethnicity, education level, occupation, economic status, pre-pregnancy height, pre-pregnancy weight, smoking status, and walking time. Pre-pregnancy BMI was calculated as pre-pregnancy weight divided by the square of pre-pregnancy height.

Anthropometric variables of each participant, including height and weight, were measured by investigators at enrollment.

According to the definition of passive smoking proposed by the World Health Organization, non-smokers who are exposed to smoke from smokers for at least 15 minutes one day per week are considered passive smokers. The Pittsburgh Sleep Quality Index (PSQI) was used to assess the sleep quality of individuals, where those with total PSQI scores ≤ 5 were classified as having good sleep quality, while those with total PSQI scores > 5 were classified as having poor sleep quality [31]. The Self-rating Anxiety Scale (SAS) was utilized to evaluate the anxiety levels of pregnant women. Scores below 50 generally indicate the absence of anxiety symptoms, whereas scores of 50 and above mean the presence of anxiety symptoms [32]. The Edinburgh Postnatal Depression Scale (EPDS) was used to assess the depression symptoms [33]. The total scores ranged from 0 to 30, with higher scores implying more severe depressive symptoms. We considered 10 as the cutoff point for determining the presence or absence of depressive symptoms.

### 2.4. Ethical Considerations

The study was approved by the Ethics Committee of Xiangya School of Public Health, Central South University (No. XYGW-2022-7, approval date: 9 March 2022). Before enrollment, informed consent was obtained from all participants.

### 2.5. Sequence and Statistical Analysis

The Student’s *t*-test was employed to compare normally distributed variables across two groups, while the Mann–Whitney U test was used for non-normal distributions. For categorical variables, Pearson’s chi-squared test or Fisher’s exact test was applied.

Binary logistic regression was used to analyze the effects of dietary patterns on GDM. The significant factors detected in the univariate analysis, energy, and other dietary patterns were added to the model for correction.

The Kruskal–Wallis test was used to compare the alpha diversity indices (Chao 1, Observed features, Shannon, and Simpson indices) of pregnant women with and without GDM. We utilized Wekemo Bioincloud (https://www.bioincloud.tech, accessed on 10 September 2023) to perform the Linear Discriminant Analysis Effect Size (LEfSe), aiming to explore the differential taxa between the two groups. In LEfSe analysis, the gut microbiota data were processed from phylum to genus levels, and linear discriminant analysis (LDA) score was set at ≥4.0. Spearman’s correlation was used to assess associations between dietary patterns and alpha diversity of the gut microbiota and differential taxa.

Mediation analysis was performed to assess the indirect effects of dietary patterns mediated by alterations in the gut microbiota with GDM. The bootstrap method was used to assess the stability of the regression model. Covariates were significant factors for GDM detected in univariate analysis, as well as other dietary patterns and energy. All statistical analysis and graph plotting were conducted in R software (version 4.1.3, R Development Core Team) and GraphPad Prism 9.5.0 (GraphPad Software, San Diego, CA, USA). Statistical tests were two-sided, considering a significance level of *p* < 0.05.

## 3. Results

### 3.1. Characteristics of Participants

A total of 185 pregnant women were included in this study, including 107 in the control group and 78 in the case group. The general characteristics of the participants are presented in Table 1. Compared to the control group, pregnant women with GDM had a higher pre-pregnancy BMI (*p* < 0.001). There was a higher proportion in the case group, which was pregnant women with ≥1 pregnancies and passive smoking (*p* = 0.004; *p* = 0.021). There were no significant differences in age, gestational weeks, weight gain during pregnancy, education level, employment status, household income status, walking time, sleep quality, anxiety symptoms, and depression symptoms.

### 3.2. Dietary Pattern Extraction

Based on distinct inflection points in the scree plot (Figure S1), eigenvalues greater than 1, cumulative variance contribution, and interpretability of dietary patterns, we extracted five dietary patterns from the data of 185 women who completed the FFQ. The first dietary pattern identified was defined as “processed food”, consisting of beverages, snacks, and processed meat products. The second pattern was labeled “meat” and consisted of poultry and animal meats. The main components of the third pattern were mushrooms, seaweeds, and beans. The fourth pattern, labeled “cereals and potatoes-eggs and milk”, was characterized by a predominance of cereals, potatoes, eggs, and milk. The fifth dietary pattern, “vegetables-fruits”, covered mainly vegetables and fruits. The percentage of variance explained by each dietary pattern during pregnancy was 15.1%, 12.2%, 9.9%, 9.2%, and 8.6%, respectively. A total of 55.1% of the variance was explained by the five dietary patterns. The eigenvalues for each dietary pattern were 1.97 (processed food), 1.59 (meat), 1.28 (fungi and algae-beans), 1.20 (cereals and potatoes-eggs and milk), and 1.12 (vegetables-fruits).

### 3.3. The Association between Dietary Patterns and GDM

To investigate the relationship between dietary patterns and GDM, the median of the dietary pattern factor scores was used as a cutoff value to categorize the dietary patterns into low and high groups. The comparison results of the two groups’ dietary patterns scores are shown in Table S3, indicating significant differences only in vegetables-fruits dietary patterns between the two groups. The proportion of pregnant women with GDM whose factor scores for the vegetables-fruits dietary pattern were in the high group (factor scores ≥ –0.07) was lower than in the control group (*p* = 0.001), and significant results from the Chi-square test will be included in further analysis (Table S4).

Binary logistic regression analysis was used to establish the model. The results revealed that in the vegetables-fruits dietary pattern, pregnant women with factor scores ≥ 0.07 had a lower risk of GDM compared to those with factor scores < 0.07 (OR = 0.32; 95% CI: 0.17–0.61; *p* < 0.001), and the significance remained after adjusting for a range of factors (Table 2).

### 3.4. Comparison of Alpha Diversity and Differential Taxa in the Two Groups

**Figure 1:**
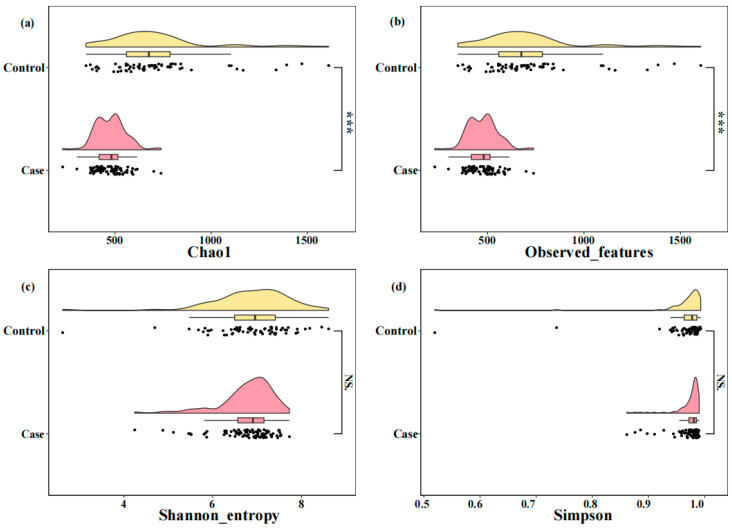
Comparison of the Alpha diversity indices of the gut microbiota of pregnant women in two groups; *** *p* < 0.001, NS means the difference is not statistically significant. (**a**) Chao1; (**b**) Observed features; (**c**) Shannon_entropy; (**d**) Simpson.

**Figure 2:**
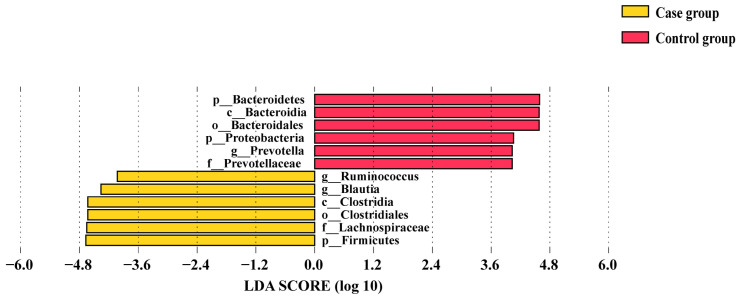
Histogram of the distribution of LDA values of the gut microbiota in the two groups: p: phylum; c: class; o: order; f: family; g: genus.

A total of 153 fecal samples were collected, 88 from the case group and 65 from the control group. The rarefaction curve indicated that the sample size was adequate (Figure S2). In terms of species richness, both the Chao1 indices and observed features of the case group were significantly lower than those of the control group (*p* < 0.001, Figure 1a,b). However, considered from the point of view of species diversity, no significant differences were observed between the two groups when assessing the Shannon and Simpson indices (Figure 1c,d). Differences in gut microbiota composition between the GDM group and control subjects were assessed at five different taxonomic levels, including phylum, class, order, family, and genus. At each level, the top 10 taxa based on relative abundance were presented (Figure S3). LEfSe analysis was used to identify differential flora in the two groups, and the results are presented in Figure 2. On the phylum level, the GDM group was enriched in Firmicutes, while the control group showed enrichment in Bacteroidetes and Proteobacteria. On the class level, the GDM group exhibited enrichment in Clostridia, whereas the control group showed enrichment in Bacteroidia. On the order level, the GDM group was marked by Clostridiales, whereas the control group was enriched in Bacteroidales. On the family level, the GDM group exhibited a predominance of the Lachnospiraceae family, while the control group showed enrichment in the Prevotellaceae family. On the genus level, the GDM group was enriched in *Blautia* and *Ruminococcus*, whereas the control group was enriched in *Prevotella*.

### 3.5. Association between Vegetables-Fruits Dietary Pattern and Gut Microbiota

**Figure 3:**
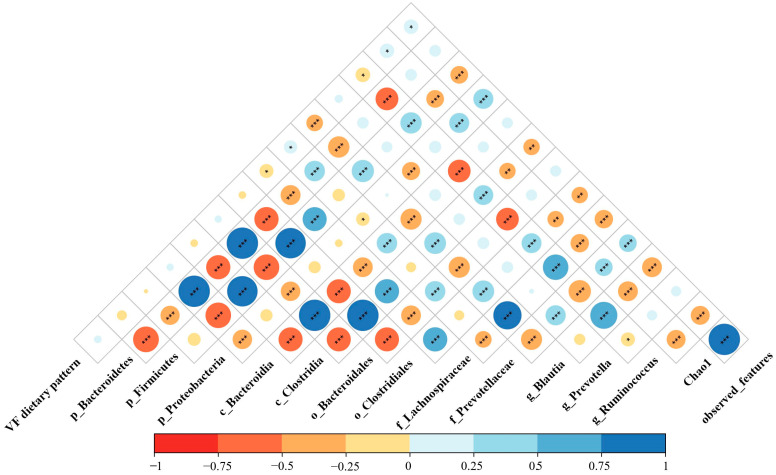
Heatmap of the correlation between different bacterial taxa and the vegetables-fruits dietary pattern. Blue means positive correlation, and red means negative correlation. VF: vegetables-fruits. *** *p* < 0.001, ** *p* < 0.01, * *p* < 0.05.

Spearman’s rank correlation analysis was used to explore the relationship between vegetables-fruits dietary patterns and gut microbiota, and the results are presented in Figure 3. Chao1 and observed features were positively correlated with vegetables-fruits dietary pattern factor scores. Among the 12 differential taxa found in the LEfSe analysis, vegetables-fruits dietary pattern factor scores were significantly negatively correlated with the Lachnospiraceae family, *Blautia*, and the *Ruminococcus* genus and were positively correlated with the Prevotellaceae family.

### 3.6. Mediation Effects of Differential Taxa on the Association between the Vegetables-Fruits Dietary Pattern and GDM

**Figure 4:**
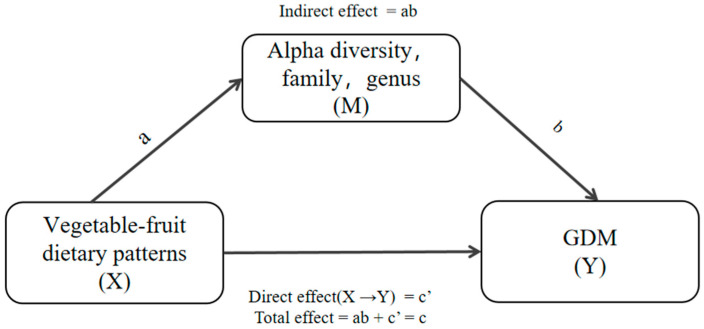
Model: the relation between vegetables-fruits dietary pattern and GDM through alpha diversity, family, and genus; GDM, gestational diabetes mellitus.

To further explore the potential mechanisms between the “vegetables-fruits” dietary pattern and GDM, mediation analysis was performed (Figure 4). After accounting for significant factors identified in the univariate analysis and energy, it was found through the mediation analysis that the gut microbiota partially mediated the relationship between vegetables-fruits dietary pattern and GDM (Table 3). In this study, three bacterial taxa (Lachnospiraceae family, *Blautia*, and *Ruminococcus* genera) were identified as mediators in the relationship between the vegetables-fruits dietary pattern and GDM. The relative abundances of the Lachnospiraceae family, *Blautia,* and the *Ruminococcus* genera, in their pathways, accounted for 40.17%, 39.15%, and 25.21% of the correlation between vegetables-fruits dietary pattern and GDM, respectively. Nevertheless, this study did not find a significant difference in the indirect effect of the alpha diversity indices. Mediation analysis revealed that two alpha diversity indices (Chao 1, Observed features) also exhibited mediating effects, with a mediation ratio of 31.91% and 32.05%.

## 4. Discussion

Findings from this study suggested that vegetables-fruits dietary pattern during pregnancy were associated with changes in the gut microbiota and reduced the risk of GDM by 67%. Through further analysis, we found that alterations in gut microbiota may partially mediate the impact of vegetables-fruits dietary pattern on GDM. These findings emphasize the potential role of gut microbiota in the prevention or treatment of GDM induced by diet.

Our findings for the vegetables-fruits dietary patterns are consistent with the majority of previous research. A study conducted in China revealed an association between a dietary pattern characterized by vegetables and a lower risk of gestational diabetes mellitus (GDM). A decrease of one quartile in the dietary pattern factor score of vegetables was associated with a 6–9% decrease in the risk of GDM [34]. In a multi-ethnic Asian cohort study, researchers pointed out that increased intake of vegetables-fruits-rice among Chinese participants was associated with a lower risk of GDM [35]. Previous studies have found that vegetables and fruits have lower energy density and glycemic load and higher levels of antioxidants and phytochemicals, all of which are protective factors against GDM [36,37,38,39]. Antioxidants may improve glucose metabolism by reducing glucose absorption, increasing insulin secretion, and improving insulin sensitivity [40]. Additionally, dietary fiber may decrease inflammation markers and regulate blood glucose levels [41].

Unlike previous studies [42,43], we did not find an association between meat dietary patterns and GDM. The divergent results may be due to the categorization of aquatic products into the meat dietary pattern in the principal component analysis, thus, masking the true effect of the meat dietary pattern. Aquatic products are rich in unsaturated fatty acids, which have been previously associated with a negative correlation with GDM [44,45]. It is worth noting that we did not find an association between a processed food dietary pattern and GDM. Processed food dietary patterns are typically characterized by high sugar and salt content, which are known to have adverse effects on blood glucose control [46]. The lack of observed effects could be attributed to the relatively modest sample size and the comparatively lower statistical power in this study.

Furthermore, our study findings indicate that the alpha diversity in GDM pregnant women differs from that of those in non-GDM. Alpha diversity is often considered a marker of health, specifically in relation to metabolic health in adults [47]. Previous studies have frequently reported a decrease in alpha diversity among GDM women [22,23,48,49], indicating a potential association between decreased alpha diversity and elevated blood glucose levels. In this study, the case group was characterized by an enrichment of five bacterial taxa within the Firmicutes phylum. The increased abundance of Firmicutes can enhance the metabolism of carbohydrates such as fructose, lactose, mannitol, starch, and sucrose, leading to elevated blood glucose levels [50,51,52].

As reported previously, a diet primarily consisting of foods rich in dietary fiber and polyphenols, such as vegetables and fruits, can improve glucose homeostasis by regulating the composition and abundance of gut microbiota [53]. There are several persuasive reasons to explain this phenomenon. Firstly, dietary fiber can be fermented into short-chain fatty acids (SCFAs) by gut microbiota [54,55,56]. The vast majority of SCFAs can be absorbed. Then, they can affect systemic glucose metabolism in some ways, including increasing satiety by acting on the gut-brain axis to suppress appetite and enhancing insulin secretion from islet β cells [54,57]. Secondly, polyphenols are common complex secondary metabolites found in plants. They can regulate glucose homeostasis by increasing glucose tolerance, reducing oxidative stress, and modulating gut microbiota, among other pathways [36,39].

Taken together, we further considered gut microbiota as the mediating factor and performed a mediation analysis. In this study, our research findings suggested that higher vegetables-fruits dietary pattern factor scores reduced the risk of GDM by increasing alpha diversity (Chao1, Observed features) and decreasing the relative abundance of differentiated taxa (Lachnospiraceae family, *Blautia* and *Ruminococcus* genera). This finding suggested that gut microbiota could be crucial in the occurrence and development of GDM, hinting that gut microbiota could be a potential target for diagnosing and treating GDM and also offering important insights into preventing and intervening in GDM through vegetables-fruits dietary strategies. The Lachnospiraceae family belongs to the Firmicutes phylum, characterized by thick cell walls. It has a close association with type 2 diabetes and has been observed to be significantly enriched in the intestines of women with GDM [58]. Additionally, within the Lachnospiraceae family, a symbiotic bacterium called *Fusimonas intestine (FI)* exists, which exhibits a higher abundance in diabetic patients [59]. Notably, some bacterial species within the *Ruminococcus* genus exhibit pro-inflammatory characteristics. One such example is *Ruminococcus gnavus* [60], which is closely associated with inflammatory diseases [61]. During the development of GDM, metabolic inflammation may play a crucial role in insulin resistance and impaired glucose tolerance [62]. When there is an imbalance in the gut microbiota, bacteria like *Ruminococcus gnavus* are likely to promote metabolic inflammation and induce GDM by producing pro-inflammatory mediators or disrupting intestinal barrier function. Unfortunately, not all studies have reported changes in *Ruminococcus* in GDM patients [21,63]. Moreover, most existing research has only explored the 16S rRNA amplicon level, and future studies should delve into the metabolic level to analyze human and bacterial metabolites in order to determine the mechanisms underlying the association between gut microbiota and GDM. Although the differential taxa found in many studies are not all the same, it is interesting to note that in several studies, scholars have reported an elevated abundance of *Blautia* in the GDM group [21,64,65]. In contrast to the predominant acid-producing genera in Firmicutes phylum, *Blautia* does not synthesize butyrate. Instead, it stimulates the secretion of tumor necrosis factor, a biomarker that shows a positive correlation with blood glucose levels in humans [66]. A large-scale study conducted in Guangzhou, China, revealed that *Blautia hydrogenotrophica* was more abundant in women with GDM and positively correlated with BMI [67]. The *Blautia* is reported as a dominant genus in individuals with glucose intolerance and is closely associated with high-fat diets and type 2 diabetes mellitus [68]. Nevertheless, some researchers found a negative correlation between *Blautia* abundance and delta glycosylated hemoglobin in women with GDM [69]. Therefore, more research evidence is needed to support the relationship between *Blautia* and GDM.

This study assessed the dietary status of pregnant women in Changsha and its association with GDM. It used factor analysis to construct dietary patterns, the results of which were instrumental in translating into applications in public health. For example, future research could focus on developing dietary index scores based on established dietary patterns and evaluating their application among GDM patients, providing reference guidelines for dietary recommendations in GDM management. Additionally, this study explored the mediating role of gut microbiota in the association between diet patterns and GDM, thus, establishing a preliminary hypothesis regarding the relationship among “vegetable and fruit-gut microbiota-GDM”. This also suggests that in the future, personalized dietary intervention programs could be developed based on the gut microbiota characteristics of pregnant women to more effectively prevent and manage GDM.

Nevertheless, some limitations should also be recognized when interpreting our findings. First, extrapolation of the study results may be limited because dietary habits may vary from region to region or from country to country. Second, this study was observational, and pregnant women may have recall bias when recalling their diets; therefore, the association does not prove causality. Finally, the vegetables-fruits dietary pattern consists of a variety of foods with a variety of nutrients such as vitamins and minerals. After establishing the link between the vegetables-fruits dietary pattern and GDM, additional research is required to understand how specific foods and nutrients contribute to this pattern.

## 5. Conclusions

In this study, we observed a significant association between an increase in vegetables-fruits dietary pattern factor scores during pregnancy and substantial modifications in the composition of the gut microbiota, consequently leading to a pronounced decrease in the risk of developing GDM. The five differential taxa of the gut microbiota were higher in pregnant women with GDM compared to those without GDM. Increased scores on the vegetables-fruits dietary pattern factor during pregnancy may reduce the risk of GDM through changes in the gut microbiota (Lachnospiraceae family, *Blautia*, and *Ruminococcus* genera). Given the variety of adverse effects associated with GDM, these findings have large public health implications, pointing to the possibility that the gut microbiota may be a novel idea for intervening in diet-related GDM. In the future, more studies can be conducted, including longitudinal prospective studies with large samples and multi-omics technology to explore the interaction mechanism between the three groups; and horizontally, dietary assessment can be carried out using more precise methods to explore the interaction mechanism of “diet-gut microbiota-GDM”.

## Figures and Tables

**Table 1:** Comparison of general characteristics of participants in the two groups.

	Control Group (*n* = 78)	Case Group (*n* = 107)	*p*
Age, years, M (P_25_, P_75_)	32 (29, 34)	32 (29, 35)	0.089
Gestational weeks, weeks, M (P_25_, P_75_)	24 (24, 25)	25 (24, 26)	0.052
Pre-pregnancy BMI, kg/m^2^, Mean ± SD ^#^	20.91 ± 2.35	22.65 ± 3.25	**<0.001**
Weight gain during pregnancy, kg, M (P_25_, P_75_) ^#^	6.45 (4, 8)	6.00 (5, 8)	0.375
Education level, *n* (%)			0.687
Senior middle school and below	8 (10.3)	13 (12.1)	
Associate degree	21 (26.9)	23 (21.5)	
Bachelor’s degree	41 (52.5)	55 (51.4)	
Master’s degree and above	8 (10.3)	16 (15.0)	
Employment status, *n* (%)			0.245
Unemployed	32 (41.0)	35 (32.7)	
Employed	46 (59.0)	72 (67.3)	
Household income status, RMB per month, *n* (%)			0.291
<5000	10 (12.8)	8 (7.5)	
5000~9999	19 (24.4)	35 (32.7)	
≥10,000	49 (62.8)	64 (59.8)	
Parity, times, *n* (%)			**0.004**
0	59 (75.6)	59 (55.1)	
≥1	19 (24.4)	48 (44.9)	
Passive smoking, *n* (%)			**0.021**
No	70 (89.7)	82 (76.6)	
Yes	8 (10.3)	25 (23.4)	
Walking time, min/d, *n* (%)			0.719
≤20	52 (66.7)	74 (69.2)	
>20	26 (33.3)	33 (30.8)	
PSQI score levels, *n* (%)			0.715
Good quality	49 (62.8)	70 (65.4)	
Bad quality	29 (37.2)	37 (34.6)	
Anxiety symptoms, *n* (%)			0.719
No	76 (97.4)	104 (97.2)	
Yes	2 (2.6)	3 (2.8)	
Depression symptoms, *n* (%)			0.336
No	63 (80.8)	80 (74.8)	
Yes	15 (19.2)	27 (25.2)	

M, median. BMI, body mass index; PSQI, Pittsburgh Sleep Quality Index. ^#^ indicates the presence of missing data. Bold values mean a significant difference (*p* < 0.05).

**Table 2:** Binary logistic regression model of association between dietary patterns and GDM (*n* = 185).

	*β*	Wald χ^2^	OR (95% CI)	*p*
Crude Model				
low (Ref.)				
high	0.98	10.10	0.38 (0.20, 0.68)	**0.001**
Model 1				
low (Ref.)				
high	1.08	10.14	0.34 (0.17, 0.65)	**0.001**
Model 2				
low (Ref.)				
high	−1.10	7.12	0.33 (0.15, 0.74)	**0.008**

Crude Model: no covariates were adjusted. Model 1: adjusted for age (years), gestational week (weeks), pre-pregnancy BMI (kg/m^2^), parity (times), and passive smoking status. Model 2: adjusted Model 1 and other dietary patterns and energy. Bold values denote a significant difference (*p* < 0.05).

**Table 3:** Mediating effects of alpha diversity and differential taxa on the associations of vegetables-fruits dietary patterns with GDM.

Mediators	Indirect Effect			Direct Effect			Proportion Mediated (%)
	*β*	*SE*	Boot 95% CI	*β*	*SE*	Boot 95% CI	
Lachnospiraceae family	0.094	0.040	−0.175, −0.018 *	−0.140	0.064	−0.265, −0.020	40.17
Prevotellaceae family	−0.024	0.017	−0.061, 0.001	−0.210	0.078	−0.359, −0.065	10.26
*Blautia* genus	−0.092	0.037	−0.177, −0.033 *	−0.143	0.073	−0.283, −0.006	39.15
*Ruminococcus* genus	−0.059	0.022	−0.126, −0.022 *	−0.175	0.069	−0.314, −0.041	25.21
Chao1	−0.075	0.035	−0.148, −0.010 *	−0.160	0.065	−0.292, −0.039	31.91
Observed features	−0.075	0.035	−0.142, −0.009 *	−0.159	0.063	−0.280, −0.040	32.05

* means the significance of the indirect effects.

## Data Availability

The data that support the findings of this study are not publicly available, due to the data containing information that could compromise the participants’ privacy, but are available from the corresponding author upon reasonable request.

## Supplementary Materials

The following supporting information can be downloaded at https://www.mdpi.com/article/10.3390/nu16142300/s1, Table S1. Factor loading matrix after rotation; Table S2. Participants’ dietary pattern factor scores; Table S3. Dietary patterns factor scores of pregnant women in the two groups, M (P25, P75); Table S4. Distribution of dietary patterns factor scores of pregnant women in the two groups; Figure S1. Scree plot; Figure S2. Rarefaction curves of observed features index of pregnant women in two groups; Figure S3. Comparison of the top 10 abundances of gut microbiota between two groups.
