# Supplementary material for: Association of Vegetables-Fruits Dietary Patterns with Gestational Diabetes Mellitus: Mediating Effects of Gut Microbiota
Source: Nutrients. 2024 Jul 17;16(14):2300. doi: 10.3390/nu16142300 (PMC11279562; doi:10.3390/nu16142300)
Supplement: Supplementary file 1 [file nutrients-16-02300-s001.zip › nutrients-3083290-supplementary.pdf]

Association of dietary patterns with gestational diabetes mellitus: mediating effects of gut microbiota

Supplementary Materials:

Table S1. Factor loading matrix after rotation

Food types	Processed food	Meat	Fungi and algae-beans	Cereals and potatoes-eggs and milk	Vegetables-fruits
Beverages	**0.78**	0.02	−0.05	−0.15	−0.15
Snacks	**0.61**	0.16	−0.17	−0.02	0.21
Processed meat products	**0.59**	−0.30	0.29	0.00	−0.09
Poultry	0.23	**0.75**	0.09	0.13	0.12
Animal meats	−0.25	**0.75**	0.05	−0.11	−0.12
Aquatic products	0.08	**0.45**	0.20	0.43	0.27
Beans	0.05	0.03	**0.75**	0.17	−0.09
Mushrooms and seaweeds	−0.11	0.16	**0.71**	−0.11	0.14
Eggs	−0.20	−0.03	0.19	**0.69**	−0.02
Cereals and tubers	−0.05	−0.03	0.28	**−0.59**	0.35
Milk and dairy products	−0.06	0.08	−0.04	**0.52**	0.50
Fruits	0.19	0.06	−0.07	−0.01	**0.65**
Vegetables	−0.22	−0.02	0.12	−0.08	**0.58**

Bold values mean the absolute value of the factor loadings > 0.4.

Table S2. Participants' dietary pattern factor scores

Num	Group	Processed food	Meat	Fungi and algae-beans	Cereals and potatoes-eggs and milk	Vegetables-fruits
1	2	5.60	3.43	−0.69	−0.23	−0.08
2	1	−0.63	−0.91	−0.28	1.34	0.02
3	1	−0.49	−0.96	−0.82	0.45	−0.93
4	2	6.57	−1.46	−0.17	−0.32	−1.70
5	2	−0.17	−0.85	0.38	−0.22	0.27
6	1	1.20	1.01	−0.13	−0.33	2.12
7	2	−0.26	−0.76	−0.60	0.08	−0.32
8	1	−0.54	−0.39	0.50	3.02	0.15
9	2	−0.61	−0.30	−0.46	0.43	−0.07
10	2	0.13	−0.22	−0.72	−1.63	0.91
11	2	−0.31	0.49	−0.51	−0.60	−1.77
12	2	−0.02	1.07	0.57	−0.45	0.03
13	2	0.36	1.05	0.84	−1.34	−0.41
14	1	0.90	−1.43	0.78	−0.18	0.24
15	1	−0.38	−0.29	−1.15	−0.13	−0.59
16	1	−0.23	−0.34	3.27	−2.51	0.59
17	2	−0.82	−1.57	0.63	−1.28	1.51
18	2	4.78	−0.18	−1.17	−1.35	−1.00
19	1	−0.32	−0.40	−0.14	−0.51	0.12
20	2	0.62	−0.79	0.11	−0.24	0.19

21	2	0.08	−0.92	−0.32	1.08	−1.19
22	2	−0.07	0.01	−0.42	−1.18	−1.21
23	1	−0.51	0.45	−0.45	−1.16	−0.49
24	1	−0.20	−0.64	−0.39	0.79	−0.41
25	1	0.43	1.81	0.72	2.15	2.21
26	2	1.11	0.30	−0.47	−0.02	0.38
27	2	−0.47	0.36	0.21	−0.48	1.42
28	1	0.06	0.34	0.86	−0.42	0.87
29	1	−0.41	0.63	−1.47	0.06	−1.48
30	1	−0.80	−0.05	−0.68	−0.74	0.87
31	1	−0.38	0.23	0.29	−0.49	1.05
32	2	−0.48	0.25	−0.65	−0.53	−2.08
33	1	−0.66	−0.22	−1.30	0.55	0.76
34	1	1.47	0.68	−1.14	−0.66	0.82
35	2	0.60	−1.05	−0.17	−0.68	−0.55
36	2	−0.60	−0.82	−0.58	0.99	0.36
37	2	0.40	0.57	1.31	−1.26	0.07
38	2	0.40	2.13	−0.27	−0.20	−0.30
39	1	1.67	−0.19	−0.05	−0.28	−0.18
40	2	1.09	−1.38	2.17	−0.15	−0.40
41	2	−0.70	−0.37	0.97	1.50	−1.12
42	2	−0.60	0.51	−1.41	0.37	−0.21
43	2	−0.90	0.68	−0.26	−0.98	−1.47
44	2	0.80	−0.63	−1.17	0.02	1.54
45	2	0.40	−0.71	−0.94	0.43	0.55
46	2	−1.00	0.32	−0.21	0.37	−1.60
47	2	−0.39	0.24	−0.24	1.63	−1.06
48	2	0.03	0.68	−0.37	0.71	0.59
49	1	0.00	−0.22	−1.08	−1.11	−0.81
50	1	−0.69	−0.43	−0.36	−0.56	−0.45
51	1	−0.52	0.26	1.89	−0.01	0.65
52	1	−0.29	0.02	0.90	0.29	−0.88
53	2	0.80	0.55	−0.77	−0.30	0.64
54	2	2.98	−1.05	1.67	−0.77	−0.46
55	2	−0.14	−0.55	1.33	2.06	−1.43
56	2	−0.30	−0.05	−0.57	1.11	−0.42
57	2	−0.75	−0.59	0.27	0.49	−0.67
58	2	−0.58	−0.29	−0.24	−0.60	−0.24
59	2	−0.68	−0.22	−0.23	−1.00	−0.98
60	1	−0.62	−1.08	0.56	−0.17	1.62
61	2	−0.32	0.11	0.01	−1.48	−0.80
62	1	−0.85	−0.61	3.76	−0.20	−0.52
63	1	−0.39	0.73	−0.69	−1.87	0.06
64	1	−0.59	0.41	1.23	0.54	0.93
65	2	−0.47	−1.19	−0.19	0.26	−0.10
66	2	−0.81	−0.50	−0.68	0.21	0.66

67	1	−0.15	0.63	−0.28	−0.37	−1.31
68	2	0.62	−1.16	3.67	−0.90	0.12
69	1	−0.63	1.45	−0.74	−2.07	−0.60
70	2	−0.37	−0.27	−0.52	0.06	0.82
71	2	1.67	−2.22	0.63	0.29	−0.23
72	2	−0.22	1.41	−0.84	−0.92	−0.62
73	2	−0.70	0.51	0.55	0.39	−1.19
74	2	−0.56	−0.09	1.81	−0.12	−0.77
75	2	0.07	−0.27	−0.55	0.23	−1.63
76	1	−0.92	−0.17	−0.20	−0.38	−1.43
77	2	−0.32	0.93	0.20	−0.99	−0.77
78	2	−0.35	−0.36	−0.95	0.39	−0.18
79	2	−0.91	−0.20	0.56	−0.73	−0.78
80	2	0.21	−0.83	0.03	0.14	−0.47
81	2	−0.45	−0.56	−0.31	0.71	0.82
82	1	−0.33	−0.77	−0.25	0.36	0.46
83	1	−0.85	−0.82	0.34	−1.23	0.00
84	2	−0.01	−1.05	−0.68	0.74	0.47
85	2	−0.16	−0.13	−0.17	2.39	−0.94
86	1	−0.85	−0.21	−0.11	1.19	−0.85
87	2	0.11	2.25	−0.42	−0.57	−0.26
88	2	−0.23	0.70	−1.26	0.51	−0.15
89	1	−0.23	−1.19	0.03	−0.66	0.27
90	2	0.12	1.45	−0.44	−0.33	−1.35
91	2	−0.90	−0.71	−0.03	−0.04	0.03
92	1	−0.49	−0.30	−0.84	−0.25	0.40
93	2	0.22	−0.70	−0.37	−0.43	−0.25
94	1	0.47	1.36	0.03	−0.03	−1.30
95	1	0.47	0.33	−0.54	1.75	0.32
96	2	0.00	0.25	−0.76	−0.25	0.71
97	2	−0.30	−0.27	1.29	0.17	−0.69
98	2	−0.44	0.23	1.04	0.27	−0.72
99	2	−0.77	1.70	−0.37	−0.41	−0.37
100	2	−0.40	0.90	−0.05	−0.93	0.40
101	2	−0.40	0.44	0.90	0.22	−1.15
102	1	−0.62	0.71	2.22	0.72	−0.51
103	2	0.33	−1.35	0.42	0.22	−0.62
104	2	0.23	−0.51	−0.62	0.24	−0.17
105	1	−0.95	1.09	1.01	−1.50	0.07
106	1	−0.03	−0.19	3.16	−2.35	0.02
107	1	0.17	0.40	−0.14	4.08	1.49
108	1	0.07	−0.73	−0.67	1.21	0.17
109	1	−0.40	3.48	1.31	0.75	0.65
110	1	−1.00	2.02	0.24	−1.42	−0.17
111	1	0.40	−1.23	−0.58	0.12	0.42
112	2	−0.60	−0.37	−0.20	−0.08	−0.36

113	2	0.03	−0.15	−1.88	1.71	0.98
114	2	−1.19	0.15	0.13	0.23	0.75
115	2	0.56	−0.39	0.46	0.47	−0.86
116	1	−0.36	0.93	−0.09	0.34	1.62
117	2	−0.20	0.14	0.29	0.00	1.94
118	1	0.00	0.01	−0.64	0.60	1.07
119	2	−0.88	−0.22	−0.54	−0.44	−1.32
120	1	−0.06	−0.35	−1.19	1.86	0.13
121	2	0.47	4.29	2.84	0.59	1.15
122	2	0.27	−0.66	0.57	0.68	−1.00
123	1	−1.64	−1.36	−0.49	−1.82	4.80
124	2	−0.43	−0.55	−0.31	0.57	0.24
125	2	−0.75	0.75	0.25	−0.96	0.68
126	1	0.50	1.74	0.14	0.77	1.69
127	1	−0.37	−0.72	−0.97	0.22	−1.35
128	2	−0.61	0.03	−0.66	0.07	−0.59
129	2	−0.63	−0.94	−0.54	−0.45	−0.42
130	1	−0.94	−0.18	0.52	−0.91	−1.75
131	1	−0.21	−0.29	0.17	−0.50	−0.90
132	1	−0.64	−0.03	−1.26	−1.06	−0.72
133	2	−0.18	1.00	−1.00	1.53	−1.01
134	2	0.30	1.76	−1.05	−0.14	0.86
135	1	0.38	−0.84	−0.89	0.68	1.18
136	1	−0.51	0.41	−0.19	0.62	0.54
137	1	−0.50	−0.14	2.54	0.86	−0.47
138	2	1.23	0.41	−1.49	0.59	−0.66
139	2	−0.46	0.87	0.67	0.43	0.89
140	1	0.46	1.14	−0.32	−0.27	0.79
141	2	−0.54	−0.89	−0.56	0.59	0.19
142	2	2.37	0.74	−0.99	−1.08	2.05
143	2	−0.35	−1.08	−0.36	0.53	1.57
144	1	0.11	−0.37	0.13	0.34	0.33
145	2	2.05	−1.80	4.31	2.26	−0.76
146	1	−0.21	−1.04	−0.73	−0.26	1.48
147	1	−0.11	−0.08	−0.45	−0.02	−0.02
148	2	0.13	1.41	−0.32	0.08	−0.61
149	1	0.86	−0.14	0.12	−1.14	0.77
150	1	0.46	0.20	1.62	−1.16	−0.04
151	2	−0.12	−0.09	−0.66	0.11	1.20
152	2	1.01	−0.37	−0.44	0.03	−0.83
153	2	1.02	−0.69	0.39	0.76	1.23
154	2	0.20	1.75	1.30	1.28	1.89
155	1	−0.08	−0.63	0.24	0.42	0.67
156	2	0.09	0.76	0.36	−0.88	0.78
157	1	−0.15	−0.91	−1.09	0.59	−0.57
158	2	0.63	1.50	0.05	3.21	0.19

159	1	−0.61	0.65	−1.36	−0.73	−1.18
160	1	−0.76	−0.43	0.34	0.74	0.87
161	1	0.34	−1.35	−0.32	−1.52	1.39
162	2	−0.78	0.06	−0.63	−0.22	−1.12
163	1	−0.47	−0.65	0.55	0.06	−0.55
164	2	−0.19	−0.71	1.13	0.97	−0.22
165	1	−0.51	−0.77	−0.44	1.33	−0.06
166	2	−0.55	−0.48	0.08	−0.37	−0.47
167	1	0.45	−0.35	−0.80	0.08	0.44
168	2	−0.36	−0.23	−0.76	1.53	0.43
169	1	0.50	−0.92	−0.34	−0.86	0.28
170	1	−0.22	−0.26	0.12	0.24	−0.14
171	1	0.38	−0.48	−0.90	−1.30	0.25
172	1	−0.08	−0.87	0.05	−1.38	3.10
173	1	−0.22	1.06	−0.18	−0.62	−0.14
174	2	−0.47	−0.88	−0.84	−0.76	0.85
175	2	0.19	0.19	0.87	1.20	−0.38
176	2	2.11	−1.09	−0.46	−0.16	−0.07
177	2	1.12	−1.77	−0.69	−0.31	0.81
178	2	−0.74	−0.21	−0.18	−0.47	−1.10
179	2	1.10	−0.17	−0.52	0.27	0.08
180	1	−0.71	0.16	0.35	0.32	−0.50
181	1	−0.68	−0.90	−0.12	−1.73	1.25
182	1	0.00	0.49	0.79	−0.11	0.25
183	2	0.02	−0.63	−0.02	−0.11	−1.96
184	2	−0.84	1.65	−1.08	−1.77	−1.81
185	2	−0.19	3.47	0.19	1.28	0.29

Table S3. Dietary patterns factor scores of pregnant women in the two groups, M (P_{25}, P_{75})

	**Control group (*n* = 78)**	**Case group (*n* = 107)**	***P***
Processed food	−0.30 (−0.61, 0.08)	−0.18 (−0.54, 0.36)	0.091
Meat	−0.20 (−0.66, 0.42)	−0.20 (−0.70, 0.55)	0.841
Fungi and algae-beans	−0.14 (−0.67, 0.39)	−0.26 (−0.62, 0.38)	0.662
Cereals and potatoes-eggs and milk	−0.15 (−0.77, 0.56)	0.03 (−0.46, 0.51)	0.258
Vegetables-fruits	0.16 (−0.50, 0.80)	−0.25 (−0.80, 0.47)	**0.007**

Bold value denotes a significant difference ($P < 0.05$).

Table S4. Distribution of dietary patterns factor scores of pregnant women in the two groups (n, %)

	**Control group (*n* = 78)**	**Case group (*n* = 107)**	***P***
Processed food			
low	44(56.4)	50(46.7)	0.193
high	34(43.6)	57(53.3)	
Meat			
low	39(50.0)	53(49.5)	0.950

high	39(50.0)	54(50.5)	
Fungi and algae-beans			
low	35(44.9)	58(54.2)	0.210
high	43(55.1)	49(45.8)	
Cereals and potatoes-eggs and milk			
low	43(55.1)	50(46.7)	0.259
high	35(44.9)	57(53.3)	
Vegetables-fruits			
low	28(35.9)	64(59.8)	**0.001**
high	50(64.1)	43(40.2)	

Bold value denotes a significant difference ($P < 0.05$).

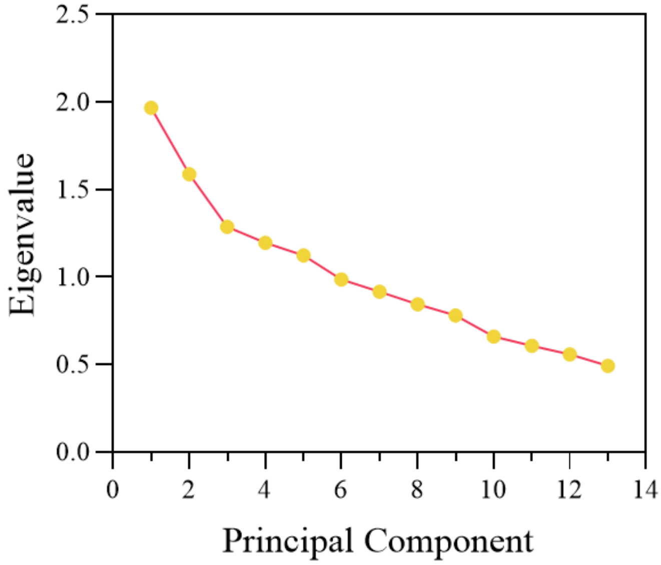

Figure S1. Scree plot

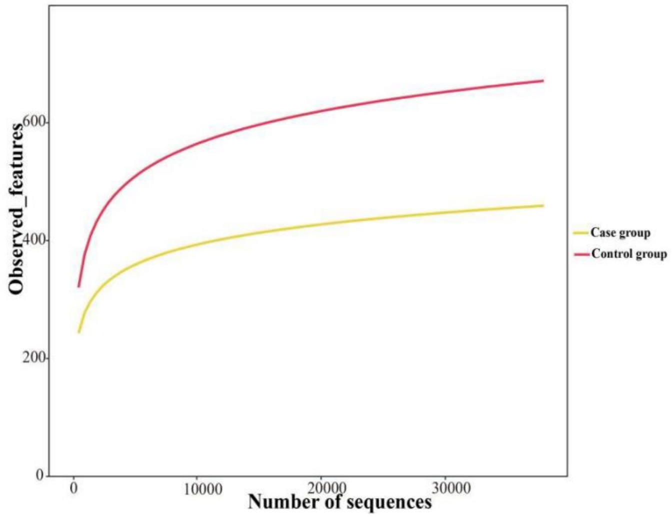

Figure S2. Rarefaction curves of observed features index of pregnant women in two groups

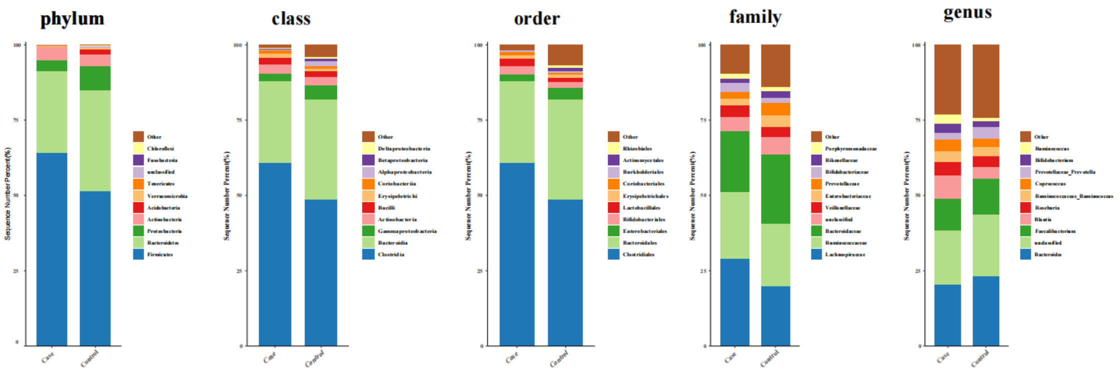

Figure S3. Comparison of the top 10 abundances of gut microbiota between two groups
